# Polar Phonon Behaviour in Polycrystalline Bi-Doped Strontium Titanate Thin Films

**DOI:** 10.3390/ma14216414

**Published:** 2021-10-26

**Authors:** Alexander Tkach, Olena Okhay, Dmitry Nuzhnyy, Jan Petzelt, Paula M. Vilarinho

**Affiliations:** 1 Department of Materials and Ceramic Engineering, CICECO–Aveiro Institute of Materials, University of Aveiro, 3810-193 Aveiro, Portugal; atkach@ua.pt (A.T.); paula.vilarinho@ua.pt (P.M.V.); 2 TEMA-Centre for Mechanical Technology and Automation, Department of Mechanical Engineering, University of Aveiro, 3810-193 Aveiro, Portugal; 3 Institute of Physics of the Czech Academy of Sciences, Na Slovance 2, 182 21 Prague, Czech Republic; nuzhnyj@fzu.cz (D.N.); petzelt@fzu.cz (J.P.)

**Keywords:** perovskites, polar dielectrics, thin films, infrared spectroscopy

## Abstract

Strontium titanate-based materials with ferroelectric or relaxor-like properties have drawn vast attention as polar dielectrics for electronics and telecommunications. Here, we study the lattice dynamics in sol–gel-derived Sr_1−1.5x_Bi_x_TiO_3_ thin films with x = 0.0053 and 0.167, deposited on Al_2_O_3_ substrates, using a variable-temperature far-infrared spectroscopy in a transmittance mode. Bi doping, known to induce a low-frequency dielectric relaxation in SrTiO_3_ (ST) ceramics and films, due to off-centre dopant ion displacements generating electric dipoles, is shown to affect the polar phonon behaviour of thin films. We show that in weakly Bi-doped films, the low-frequency polar TO1 mode softens on cooling but less than in undoped ST. In heavily Bi-doped ST films, this mode displays no significant frequency variation with temperature from 300 to 10 K. The polar phonon behaviour of polycrystalline Bi-doped ST thin films is comparable with that of Bi-doped ST ceramics, which exhibit dielectric relaxations and harden soft-mode behaviour instead of the ferroelectric phase transition.

## 1. Introduction

Perovskite-structured SrTiO_3_ (ST) stands out as an incipient ferroelectric with dielectric permittivity increasing continuously on cooling due to the polar-mode softening but without a ferroelectric phase transition [1,2,3,4,5]. Incipient ferroelectrics exhibit small values of the dissipation factor, tanδ, and strong dependence of the real part of the dielectric permittivity, ε′, on electric field, which makes them attractive for applications in tunable electronic components [6,7]. Although SrTiO_3_ has been studied for decades, its practical importance in terms of applications, in particular in the thin-film form, to respond to the most recent requirements for miniaturization from the microelectronics industry, fully justify the renewed interests in this material [8].

The lattice dynamics is of central importance for understanding the structural properties of materials. The phonon behaviour in ST bulk was studied using far-infrared (IR) spectroscopy [9], neutron scattering [10], Raman scattering [2] and hyper-Raman scattering [11]. Besides the cubic-to-tetragonal (antiferrodistortive) phase transition, observed in ST single crystals at about 110 K, due to the instability of the zone-corner soft mode [12], the soft-mode behaviour has also been shown to be the basis for the dielectric nonlinearity in ST. The zone-centre soft-mode phonon was reported to increase its frequency with applied electric field [2]. This indicates that the mechanism for the reduction of the dielectric permittivity ε′ under applied field is due to the hardening of the soft mode [2], which arises from the anharmonic restoring forces on Ti ions when displaced from their equilibrium positions [13].

According to reports on the lattice dynamics of ST films, the soft mode of the films is usually much harder than that in the bulk materials at low temperature [14,15,16,17,18,19]. Correspondingly, the phonon contribution to the low-temperature dielectric permittivity is strongly suppressed in ST films compared to bulk ST. For epitaxial films deposited by pulsed laser deposition (PLD), it was explained by influence of some specific local polar regions occurring in ST thin films during the deposition process [15]. For polycrystalline films, porosity and eccentricity of the grain boundaries are suggested as the main reasons for such behaviour [18]. Sirenko et al. reported that at high temperatures the soft-mode frequency in the film and the bulk are close at zero electric field, indicating that either the density of the local polar regions is low or the polarization of these regions is weak or small [15]. However, whereas the electric-field hardening of the soft mode vanishes in ST crystals above T ≈ 80 K [20], it persists in ST thin films until high temperatures. This fact was attributed to the polarization by the electric field of the easily polarisable local regions around oxygen vacancies, which are expected to increase the dielectric loss in thin films [15]. At the same time, Ostapchuk et al. reported that in contrast to polycrystalline ST films, a 300 nm thick ST film, quasiepitaxially grown on a (0001) sapphire substrate with a perfect (111) orientation, displays a ferroelectric phase transition near 125 K induced in the film plane by a tensile residual stress [18].

Among ST-based compounds [5], Bi-doped ST solid solutions have been intensively studied as for dielectric [21,22] as for resistance-switching memory [23] and thermoelectric applications [24]. In Sr_1−1.5x_Bi_x_TiO_3_, ceramics displacements of Bi^3+^ ions on cuboctahedral Sr sites were reported to generate local dipole moments that induce dielectric relaxations but harden the ST polar soft mode [25,26,27]. As a result, these ceramics feature no ferroelectric phase transition but relaxations at frequencies up to the THz range [27]. Similar to the Bi-doped ST ceramics, Bi-doped ST thin films exhibit dielectric relaxations, which have also been attributed to a positional disorder of Bi^3+^ on Sr sites [21,28]. 

Although the dielectric response of Bi-doped ST films on platinized Si [21,28,29,30,31,32,33,34] and polar phonon behaviour of undoped ST films on Al_2_O_3_ substrates have been investigated [18,35,36], so far no research has been undertaken of the lattice dynamics in Bi-doped ST thin films, particularly as a function of temperature. To fill this gap and determine the effect of Bi doping on the polar phonon behaviour of ST thin films, we performed a variable-temperature far-infrared spectroscopy study of sol–gel-derived Sr_1−1.5x_Bi_x_TiO_3_ thin films with x = 0.0053 and 0.167, deposited on Al_2_O_3_ substrates, since these substrates were found to not constrain the soft mode of undoped polycrystalline ST films [36].

## 2. Materials and Methods

**Figure 1:**
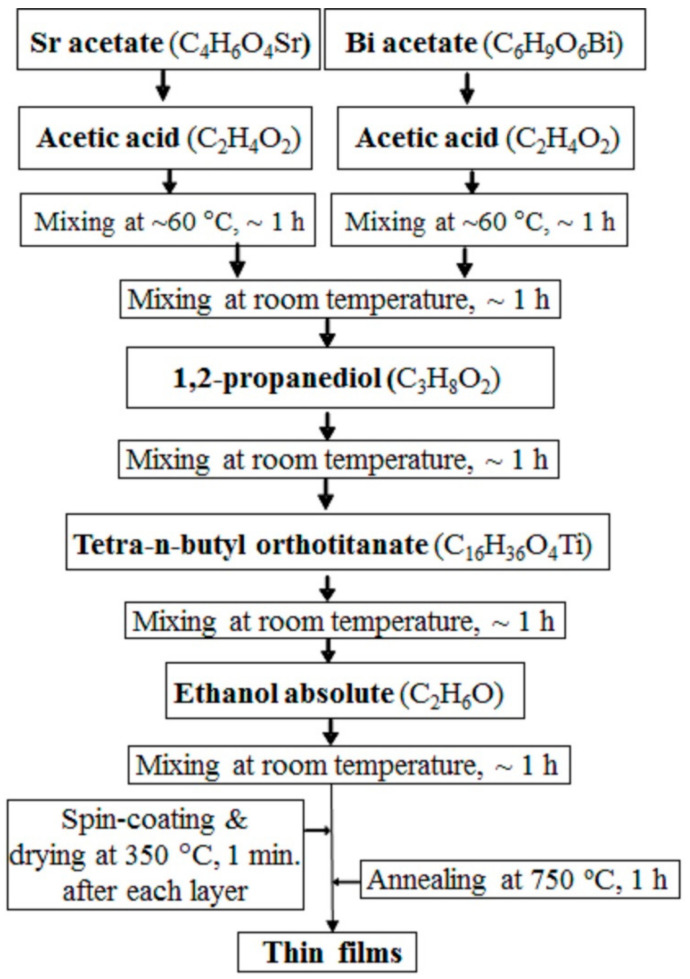
Schematic diagram of the preparation process for Sr_1−1.5x_Bi_x_TiO_3_ thin films with x = 0.0053 and 0.167 deposited on Al_2_O_3_ substrates.

For this study, we selected Sr_1__−__1.5x_Bi_x_TiO_3_ thin films with x = 0.0053 and 0.167 prepared using the sol–gel method, which has been demonstrated to yield a single perovskite phase [21,29]. For the preparation of the sols with a concentration of about 0.2 M, according to the schematic diagram shown in Figure 1, the following reagents were used in proportions depending on the film’s composition: strontium acetate C_4_H_6_O_4_Sr (98%, abcr GmbH, Karlsruhe, Germany), tetra-n-butyl orthotitanate C_16_H_36_O_4_Ti (98%, Merck KGaA, Darmstadt, Germany), bismuth acetate C_6_H_9_O_6_Bi (99%, abcr GmbH, Karlsruhe, Germany), acetic acid C_2_H_4_O_2_ (99.8%, Merck KGaA, Darmstadt, Germany), 1,2-propanediol C_3_H_8_O_2_ (99.5%, Riedel-de Haën, Seelze, Germany) and absolute ethanol C_2_H_6_O (99.8%, Merck KGaA, Darmstadt, Germany). Using the previously prepared precursor solutions, layers were deposited on the substrates by spin-coating at 4000 rpm for 30 s, using the spin coater KW-4A (Chemat Technology, Los Angeles, CA, USA). Before utilisation, the substrates were cleaned in boiling ethanol and dried on a hot plate. Subsequently, the films (substrate with the wet layer) were heated on a hot plate at 350 °C for ~1 min. This step was repeated after each spinning to ensure complete removal of the volatile species between each layer. After the deposition of the required number of layers (here 15 layers), they were annealed in air at 750 °C for 60 min with heating/cooling rate of 5 °C/min, resulting in the films with final thickness of about 450 nm. All Bi-doped ST films were deposited on double-side polished Al_2_O_3_ sapphire single crystals (Crystal GmbH., Berlin, Germany) to study them by IR spectroscopy in the transmittance mode. Similarly prepared undoped ST films were also studied for comparison. 

Compositional analysis of the films was done using an energy-dispersive spectroscopy (EDS) system (QUANTAX 75/80, Bruker, Ettlingen, Germany) in the top-view geometry under the accelerating voltage of 15 kV of a scanning electron microscope (Hitachi TM4000Plus, Tokyo, Japan). The thin-film crystal phase was analysed at room temperature in out-of-plane geometry using an X-ray diffractometer (Rigaku D/teX Ultra 250, Tokyo, Japan). The data were recorded in 0.02° step mode with a scanning rate of 1°/min from 20° to 80° using Cu Ka radiation (at 40 kV, 30 mA). Far-infrared transmittance was measured on the interferometer (Bruker IFS 113v, Ettlingen, Germany) in the ~20–250 cm^−1^ range with a resolution of 0.5 cm^−1^ in unpolarized light. Transmission geometry was chosen because it allows one to determine the TO1 mode parameters unambiguously in the case of a highly reflecting ST-based system. The spectrometer was equipped with a liquid-He-cooled bolometer used as the detector and polyethylene beam splitters of 6, 50 and 75 μm thicknesses. The samples were mounted in a helium continuous-flow Optistat CF cryostat (Oxford Instruments, Oxford, UK) with polyethylene windows and cooled down to 10 K. The incident beam was normal to the sample surface, so that only the in-plane component of the dielectric response was probed. 

## 3. Results and Discussion

**Figure 2:**
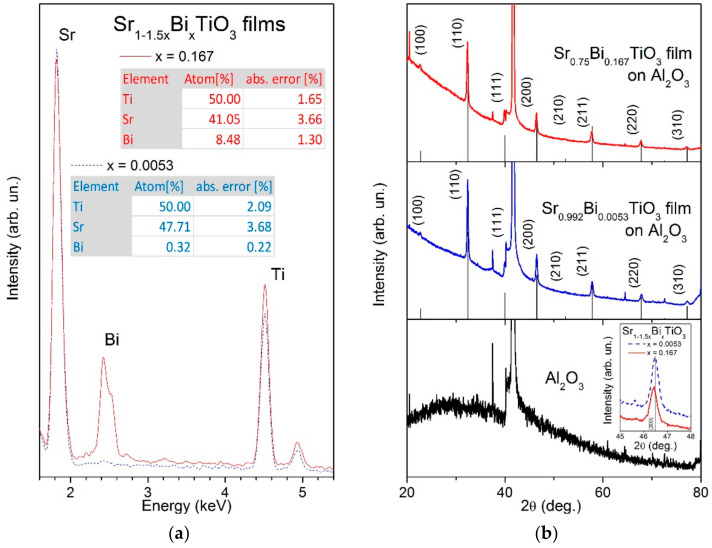
Energy-dispersive spectra and their elemental quantification results (**a**) as well as X-ray diffraction profiles (**b**) for Sr_1−1.5x_Bi_x_TiO_3_ thin films with x = 0.0053 and 0.167 deposited on Al_2_O_3_ substrates. The X-ray diffraction profile for bare Al_2_O_3_ substrate is presented in the bottom panel of (**b**). The reflections related to the perovskite structure of SrTiO_3_ (PDF#35-0734) with corresponding indexes are also shown in (**b**), as well as magnified view at reflection (200) as inset in the bottom panel of (**b**).

EDS analysis of Sr_1−1.5x_Bi_x_TiO_3_ thin films, presented in Figure 2a, clearly displays the Bi peak intensity increase with *x* value and simultaneous decrease of the Sr to Ti peak intensity ratio. Moreover, according to the spectra quantification, the bismuth concentrations in both Sr_1__−__1.5__x_Bi_x_TiO_3_ thin films with x = 0.0053 and 0.167 are very close to the nominal ones. Overall, estimated elemental contents indicate the proximity of both film compositions to the nominal ones within error bars.

X-ray diffraction (XRD) profiles of Bi-doped ST films on Al_2_O_3_ substrates are shown in Figure 2b, revealing only peaks related to the perovskite structure of SrTiO_3_ (PDF#35-0734) and those from bare Al_2_O_3_ substrate, also displayed in Figure 2b. The only difference between the weakly and heavily Bi-doped ST films is a slight shift of the XRD peaks toward lower 2θ values with Bi content, implying the lattice parameter increase. Such behaviour is in agreement with that observed in Bi-doped ST ceramics [37] and explained by the slightly larger ionic size of Bi^3+^ compared to that of Sr^2+^ at coordination number of 12 [22] as well as by the larger size of Sr vacancies present in Sr_1−1.5x_Bi_x_TiO_3_ for charge compensation compared to the size of Sr^2+^ ions [38].

**Figure 3:**
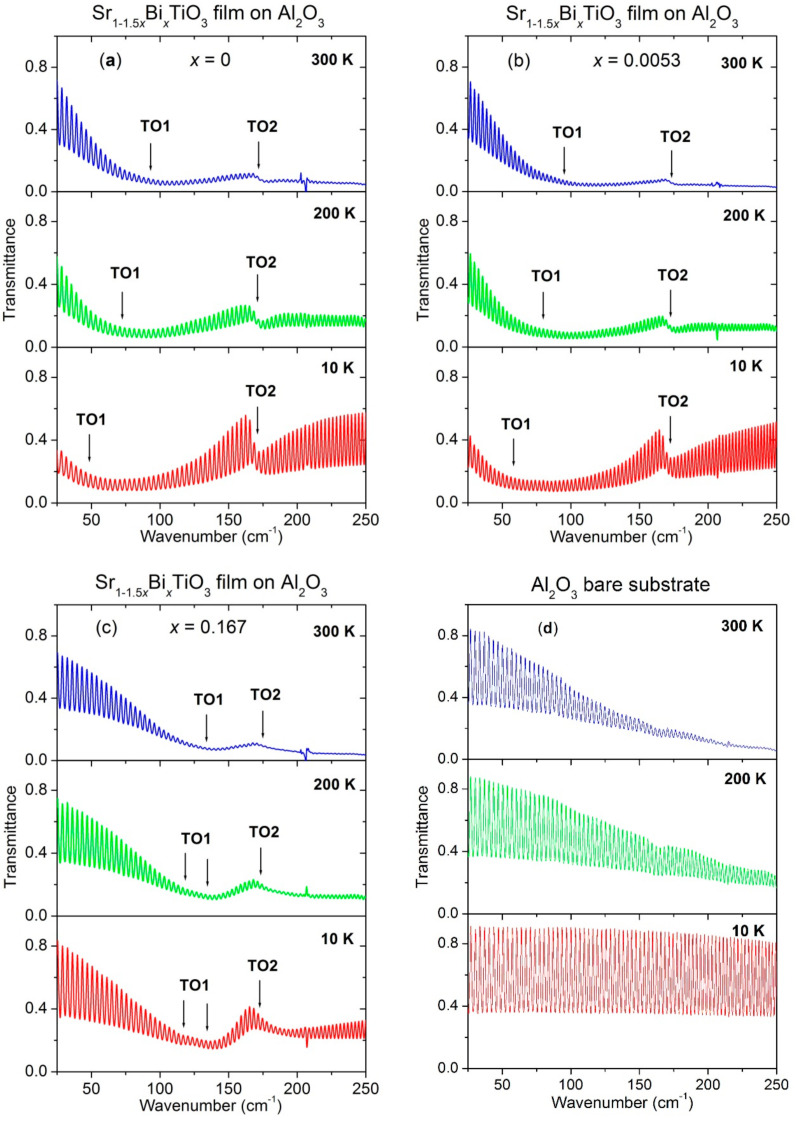
Infrared transmittance spectra at 300 K (top panel), 200 K (middle panel) and 10 K (bottom panel) of Sr_1−1.5x_Bi_x_TiO_3_ thin films with x = 0 (**a**), 0.0053 (**b**) and 0.167 (**c**) deposited on Al_2_O_3_ substrates as well as of bare Al_2_O_3_ substrate (**d**).

Figure 3 illustrates the evolution of transmittance spectra of the perovskite Bi-doped ST films deposited on Al_2_O_3_ substrates and analysed in the IR frequency range as a function of temperature from 10 to 300 K in comparison to that of undoped ST. The spectra of undoped and weakly Bi-doped ST films are rather similar, whereas those of the heavily doped ST film differ significantly, particularly in the low-frequency range. Both undoped and Bi-doped ST films show broad minimum as assigned to TO1 mode. The minimum position shifts to higher frequency with temperature and Bi content increase. A weak second minimum is also observed in the analysed films and as assigned to the TO2 phonon response, since an identical mode was reported for the ST films on Al_2_O_3_ substrates [18,36] and for the ST ceramics at the same frequency of ~176 cm^−1^ [39]. The asymmetric spectral form of TO2 suggests its coupling to the neighbouring soft mode [18].

The transmittance spectra of Sr_1−1.5x_Bi_x_TiO_3_ films (Figure 3a–c) were fitted to determine the TO mode parameters and to obtain the complex dielectric response function by the classical damped oscillator dispersion model:

(1) $$\hat{\varepsilon }(\omega )=\varepsilon^{\prime }(\omega )-i\varepsilon^{\prime \prime }(\omega )=\varepsilon (\infty )+\sum_{j=1}^{n}\Delta \varepsilon_{j}\frac{\omega_{TO_{j}}^{2}}{\omega_{TO_{j}}^{2}-\omega^{2}+i\omega \gamma_{TO_{j}}}$$

where *ε*(∞) = *n*^2^ stands for the high-frequency optical permittivity and $\omega_{TO_{j}},\gamma_{TO_{j}}$ and $\Delta \varepsilon_{j}$ denote the frequency, damping and dielectric strength of the *j*th transverse optical (*TO*) phonon mode, respectively. To reduce the ambiguity of the fit, the parameters of the substrate were obtained from an independent fit of the transmittance spectra of a bare substrate (Figure 3d) and then fixed during the subsequent fits of the transmittance spectra of films on the corresponding substrates. 

**Figure 4:**
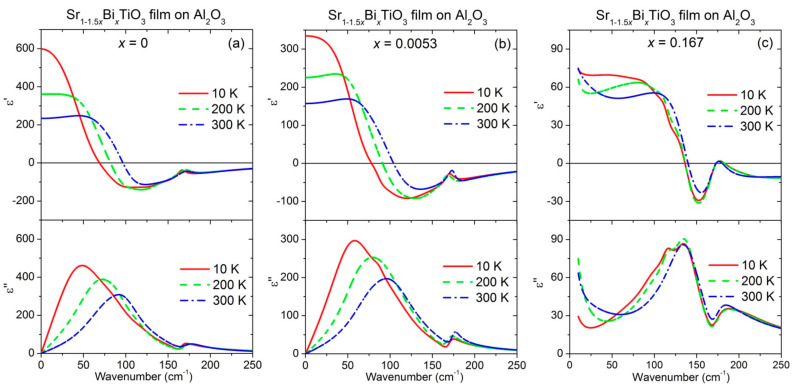
Spectra of the real *ε*′ (top panel) and imaginary *ε*″ (bottom panel) parts of the dielectric permittivity in the infrared range, obtained from the transmittance fits at 10 K (solid lines), 200 K (dash lines) and 300 K (dash-dot lines) for Sr_1−1.5x_Bi_x_TiO_3_ thin films with x = 0 (**a**), 0.0053 (**b**) and 0.167 (**c**) deposited on Al_2_O_3_ substrates.

The resulting spectra of the real *ε*′ and imaginary *ε*″ parts of the dielectric permittivity in the IR range shown in Figure 4 present a continuous decrease in the permittivity with increasing Bi content and temperature, reflecting lower polar phonon contribution to the permittivity. Correspondingly, the peak in *ε*″ spectra shifts to a higher frequency and the low-frequency permittivity decreases in agreement with the Lyddane–Sachs–Teller relation [40]. 

For undoped ST, the *ε*″ peak frequency, corresponding to TO1 mode position, varies from 48 cm^−1^ at 10 K to 72 cm^−1^ at 200 K and 91cm^−1^ at 300 K, as seen in Figure 4a. The same tendency but slightly higher frequency values of 58 cm^−1^ at 10 K, 79 cm^−1^ at 200 K and 95 cm^−1^ at 300 K can be deduced from Figure 4b for Sr_1−1.5x_Bi_x_TiO_3_ film with x = 0.0053. At the same time, the TO1 mode frequency of Bi-doped ST film with x = 0.167 is equal to ~134 cm^−1^ in the temperature range from 10 K to 300 K, as shown in Figure 4c. 

In addition, weak splitting of the TO1 soft-mode for the Sr_1−1.5x_Bi_x_TiO_3_ film with x = 0.167 is seen in Figure 4c below ~200 K. Such a feature, even if not seen in the IR reflectivity of Bi-doped ST ceramics up to x = 0.133 [27], points to the presence of anisotropic polar clusters around the Bi ions. Moreover, an increase in losses was observed at the low-frequency soft-mode wing (below 30 cm^−1^) of Sr_1−1.5x_Bi_x_TiO_3_ films with x = 0.167 (Figure 4c), not seen in the films with x = 0 and 0.0053 (Figure 4a,b). Several relaxation regions involving frequency distribution up to the THz range were reported to appear in the wide-frequency spectra of heavily Bi-doped ST ceramics [27]. These relaxations were related to the reorientation of dipoles created by the off-centred Bi^3+^ ions (individual hopping of the Bi^3+^ ions), and to the dynamics of the polar nanoclusters surrounding the Bi^3+^ ions, which interact with each other via the highly polarisable host crystal lattice (cooperative hopping of the off-centred Bi^3+^ ions). Therefore, the additional loss increase observed in Sr_1−1.5x_Bi_x_TiO_3_ films with x = 0.167 at ~10–50 cm^−1^ (Figure 4c) could have the same origin.

**Figure 5:**
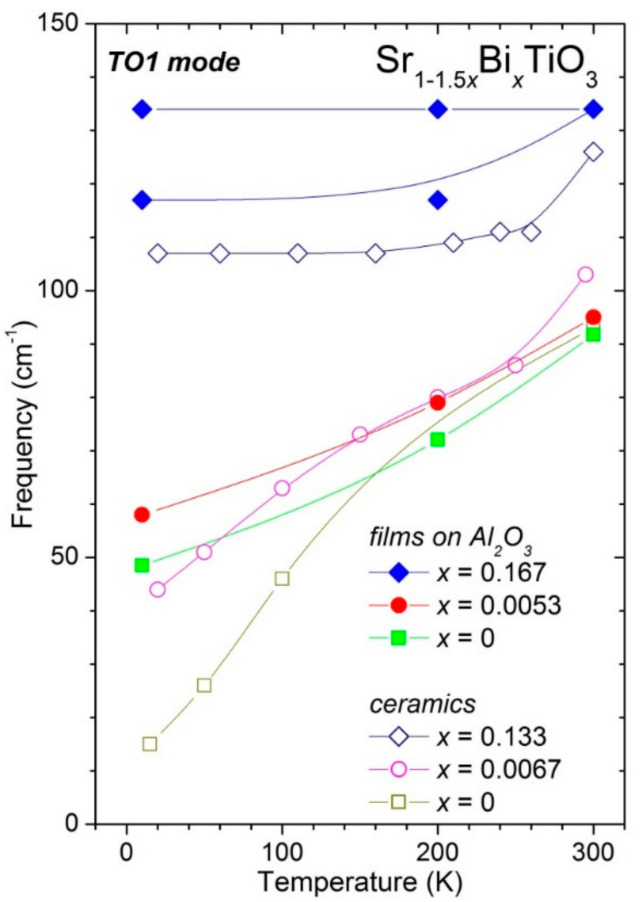
Temperature dependence of the TO1 mode frequency in Sr_1−1.5x_Bi_x_TiO_3_ thin films with x = 0, 0.0053 and 0.167 deposited on Al_2_O_3_ substrates in comparison to that of undoped and Bi-doped ST ceramics with x = 0.0067 and 0.133 [27,39]. The lines are guides for the eye.

The TO1 mode frequency values as a function of temperature are presented in Figure 5 and compared with those reported for undoped and Bi-doped ST ceramics [27,39]. As mentioned above, undoped and weakly Bi-doped ST thin films on Al_2_O_3_ substrates upon cooling display a continuous decrease of the TO1 soft-mode frequency from 91 and 95 cm^−1^ at 300 K to 48 and 58 cm^−1^ at 10 K, respectively. Similar behaviour was reported for undoped and 0.67% Bi-doped ST ceramics [27,39], as seen in Figure 5. By contrast, heavily Bi-doped ST ceramics keep the TO1 mode frequency of 134 cm^−1^ from 10 K to 300 K in agreement with the moderate mode frequency variation reported for 13.3% Bi-doped ST ceramics [27]. Thus, as in the case of Bi-doped ST ceramics, the dielectric relaxations induced by off-centre displacements of the Bi^3+^ ions on Sr sites of Sr_1−1.5x_Bi_x_TiO_3_ thin films are accompanied by reduced softening of the TO1 mode and absence of the ferroelectric phase transition. 

## 4. Conclusions

Bi doping was successfully performed in sol–gel-derived ST thin films deposited on Al_2_O_3_ substrates, as confirmed by EDS and XRD analysis, and found to have a significant effect on their phonon behaviour. In the IR transmittance spectra, the presence of polar Bi off-centring on the cuboctahedral Sr sites originating in a pronounced dielectric relaxation and the coupling of the resulting electric dipoles to the host lattice is manifested by the hardening of the low-frequency TO1 mode relative to the undoped ST. Weakly Bi-doped ST films present soft-mode behaviour, which is only slightly harder than that of undoped ST. Heavily Bi-doped ST films show a high soft-mode frequency without appreciable softening below 300 K. A comparison with Bi-doped ST ceramics, where Bi^3+^ cations also occupy Sr sites and exhibit polar displacements, indicates the similarity of such a coupling, yielding dielectric relaxations without triggering a ferroelectric phase transition.

## Data Availability

The data presented in this study are available on request from the corresponding author.
